# Supplementary material for: The effects of soleus fascicle length on muscle fatigability
Source: PeerJ. 2025 Aug 21;13:e19842. doi: 10.7717/peerj.19842 (PMC12375294; doi:10.7717/peerj.19842)
Supplement: Supplemental Information 1 — DF15: Dorsiflexed at 15 degrees; PF15: Plantarflexed at 15 degrees. [file peerj-13-19842-s001.pdf]

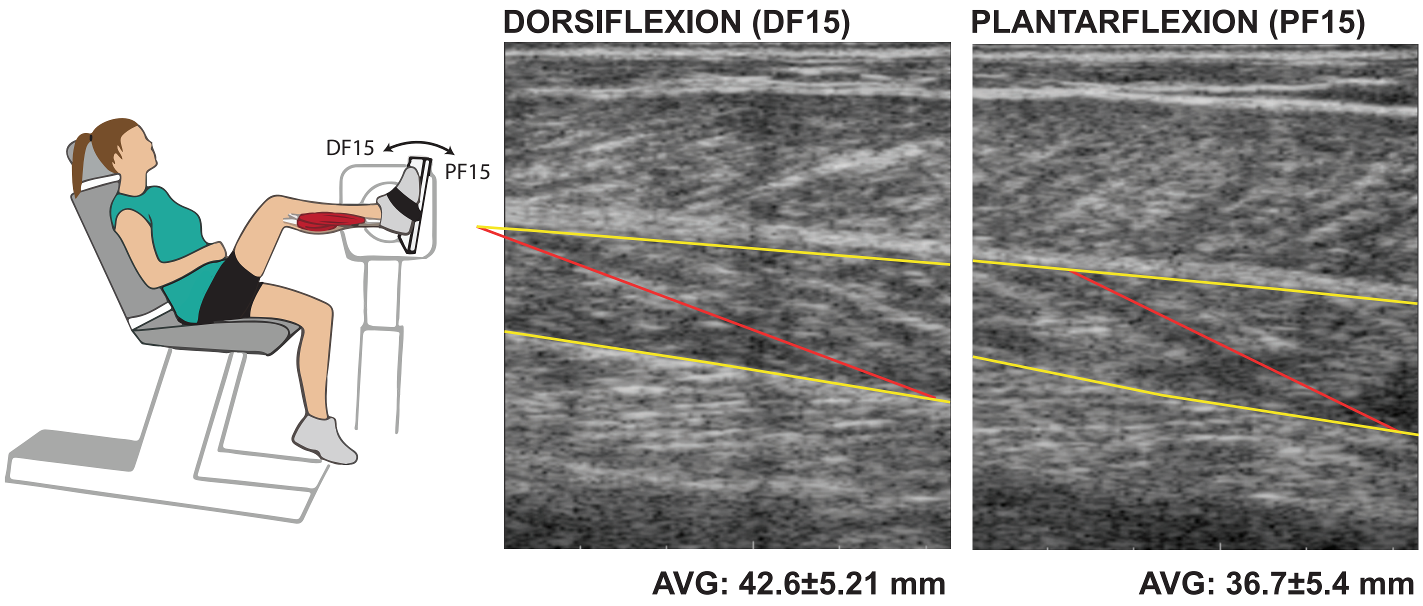

Supplementary Figure. Representative B-mode ultrasound images for a single subject showing the soleus fascicle length (with accompanying average values and standard deviations) at both ankle joint angles. Data were captured at rest and analyzed using UltraTrack.
