# Supplementary material for: The effects of soleus fascicle length on muscle fatigability
Source: PeerJ. 2025 Aug 21;13:e19842. doi: 10.7717/peerj.19842 (PMC12375294; doi:10.7717/peerj.19842)
Supplement: Supplemental Information 3 — These images provide three qualitative impressions from our data. The first, showing that the task failure was reached earlier in plantarflexion than at dorsiflexion. The second, showing that plantarflexor moment decreased over time at both postures, particularly leading up to task failure. Finally, the third, showing that rectified calf muscle excitations increased over time at both postures, particularly leading up to task failure. [file peerj-13-19842-s003.docx]

**the effects of calf muscle length on local muscle fatigability**

**SUPPLEMENTAL MATERIAL**

**
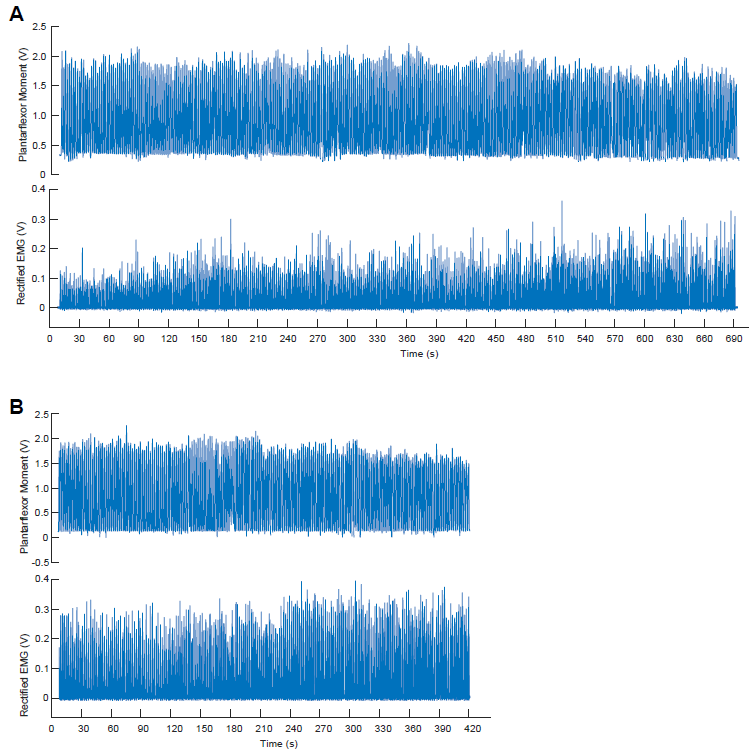
**

**Figure S1.** Time series ankle moment and rectified EMG data from a representative subject completing fixed-end contractions to fatigue at (A) 15° dorsiflexion and (B) 15° plantarflexion. These images provide three qualitative impressions from our data. The first, showing that the task failure was reached earlier in plantarflexion than at dorsiflexion. The second, showing that plantarflexor moment decreased over time at both postures, particularly leading up to task failure. Finally, the third, showing that rectified calf muscle excitations increased over time at both postures, particularly leading up to task failure.
